# The role of the peritrophic matrix and red blood cell concentration in *Plasmodium vivax* infection of *Anopheles aquasalis*

**DOI:** 10.1186/s13071-018-2752-5

**Published:** 2018-03-06

**Authors:** Djane Clarys Baia-da-Silva, Luis Carlos Salazar Alvarez, Omaira Vera Lizcano, Fabio Trindade Maranhão Costa, Stefanie Costa Pinto Lopes, Alessandra Silva Orfanó, Denner Oliveira Pascoal, Rafael Nacif-Pimenta, Iria Cabral Rodriguez, Maria das Graças Vale Barbosa Guerra, Marcus Vinicius Guimarães Lacerda, Nagila Francinete Costa Secundino, Wuelton Marcelo Monteiro, Paulo Filemon Paolucci Pimenta

**Affiliations:** 1 Diretoria de Ensino e Pesquisa, Fundação de Medicina Tropical Dr. Heitor Vieira Dourado, Manaus, AM Brazil; 2 Programa de Pós-Graduação em Medicina Tropical, Universidade do Estado do Amazonas, Manaus, AM Brazil; 3 Grupo de Investigación QUIBIO, Departamento de Biología, Universidad Santiago de Cali, Valle del Cauca, Colombia; 4 Department of Genetics, Evolution and Bioagents, Institute of Biology, Universidade Estadual de Campinas, Campinas, SP Brazil; 5 Instituto Leônidas & Maria Deane, Fiocruz, Manaus, AM Brazil; 6 Instituto de Pesquisas René Rachou, Fundação Oswaldo Cruz-Minas Gerais, Belo Horizonte, MG Brazil

**Keywords:** Malaria, *Plasmodium vivax*, Peritrophic matrix, Trypsin, Chitinase, Hematocrit

## Abstract

**Background:**

*Plasmodium vivax* is predominant in the Amazon region, and enhanced knowledge of its development inside a natural vector, *Anopheles aquasalis*, is critical for future strategies aimed at blocking parasite development. The peritrophic matrix (PM), a chitinous layer produced by the mosquito midgut in response to blood ingestion, is a protective barrier against pathogens. *Plasmodium* can only complete its life-cycle, and consequently be transmitted to a new host, after successfully passing this barrier. Interestingly, fully engorged mosquitoes that had a complete blood meal form a thicker, well-developed PM than ones that feed in small amounts. The amount of red blood cells (RBC) in the blood meal directly influences the production of digestive enzymes and can protect parasites from being killed during the meal digestion. A specific study interrupting the development of the PM associated with the proteolytic activity inhibition, and distinct RBC concentrations, during the *P. vivax* infection of the New World malaria vector *An. aquasalis* is expected to clarify whether these factors affect the parasite development.

**Results:**

Absence of PM in the vector caused a significant reduction in *P. vivax* infection. However, the association of chitinase with trypsin inhibitor restored infection rates to those of mosquitoes with a structured PM. Also, only the ingestion of trypsin inhibitor by non-chitinase treated mosquitoes increased the infection intensity. Moreover, the RBC concentration in the infected *P. vivax* blood meal directly influenced the infection rate and its intensity. A straight correlation was observed between RBC concentrations and infection intensity.

**Conclusions:**

This study established that there is a balance between the PM role, RBC concentration and digestive enzyme activity influencing the establishment and development of *P. vivax* infection inside *An. aquasalis*. Our results indicate that the absence of PM in the midgut facilitates digestive enzyme dispersion throughout the blood meal, causing direct damage to *P. vivax*. On the other hand, high RBC concentrations support a better and thick, well-developed PM and protect *P. vivax* from being killed. Further studies of this complex system may provide insights into other details of the malaria vector response to *P. vivax* infection.

## Background

Malaria remains one of the most important public health problems worldwide. Overall, it is estimated that 3.2 billion people in 97 countries and territories are at risk of being infected with *Plasmodium* species and developing the disease [1]. *Plasmodium falciparum* is most prevalent in Africa and *Plasmodium vivax* is predominant in Southeast Asia and Latin America, mainly occurring in the Amazon region [1]. Approximately 70 *Anopheles* species are considered malaria vectors in the American continent [2]. *Anopheles aquasalis* is an important malaria vector that breeds in brackish marsh [3] and is distributed predominantly along the South and Central American areas where it is considered the primary coastal malaria vector of *P. vivax* [4–6]. Furthermore, *An. aquasalis* is a species that has been colonized for several years [7] and in the last decade, it has been used as a good experimental model for interaction studies with *P. vivax* [8–13].

The peritrophic matrix (PM) is a semi-permeable and fibrous layer produced by secretion of intestinal epithelium of insects, including mosquitoes, in response to post-feeding midgut distension. The structured PM surrounds the food bolus inside the midgut and is considered responsible for several essential functions including preventing tissue damage, compartmentalization of ingested food, digestive enzyme flow control, and moreover as a protective barrier against pathogens [14]. Mosquitoes’ PM is mainly composed of chitin, proteoglycans and proteins [15]. Chitin is mainly responsible for PM mechanical and tensile strengths and glycoproteins/proteoglycans are also involved in resistance, but mainly in PM permeability [15, 16]. In haematophagous insects, distention due to blood meal ingestion stimulates midgut epithelial cells to generate a PM that remains until the end of the digestion process. In several species of mosquitoes of the genera *Aedes*, *Culex* and *Anopheles*, the PM starts to be synthetized between 3–6 h after blood ingestion and, in general, it is well structured around 24 h, persisting until around 48 h when the digestion residues are excreted from the gut [17, 18]. Interestingly, fully engorged mosquitoes that had a complete blood meal form a thicker, well-developed PM than the ones that fed in small amounts [19, 20].

The ingestion of pathogenic microorganisms, such as viruses, bacteria and protozoa, may happen during the blood-feeding of a mosquito vector on infected individuals. These microorganisms then face the PM that acts as an important barrier in the midgut. Thus, ingested pathogens, including *Plasmodium* protozoa, can only complete their life-cycle and consequently be transmitted to a new vertebrate host after successfully passing the PM barrier, the first physical barrier in the vector midgut. *Plasmodium* gametocytes, after being ingested by the mosquito vector, complete their sexual cycle in the midgut lumen and develop into ookinetes, the mobile parasite form. The ookinetes then invade the PM, leaving behind the blood bolus, and get into the surrounding midgut epithelium. *Plasmodium* parasites start to invade the PM and reach the mosquito midgut epithelial cells around 18–24 h after the infective blood meal ingestion [21–23].

Ingested *Plasmodium* faces trypsin activities, one of the major digestive enzymes that process the blood meal inside the mosquito midgut, during the gametocyte-ookinete differentiation [24]. The amount of red blood cells (RBC) in the blood meal directly influences the production of digestive enzymes [21, 22] and can protect parasites from being killed during the meal digestion [23, 25]. However, it is well known that malaria causes anemia in infected people, likely affecting physical properties of the blood meal and further PM formation [26]. Furthermore, when digestive enzymes challenge ookinetes, they are also stimulated to synthetize a typical *Plasmodium*-specific chitinase to open a hole and cross the PM [27]. Experimental studies adding substances to the infective blood meal that interfere with PM synthesis or maintain a structured PM for a longer time have provided insights into the PM role during the *Plasmodium* life-cycle inside the vector [25]. These studies were developed with *P. falciparum* and murine or avian *Plasmodium* species [25, 28, 29].

The main aim of this work was to understand the roles of the PM and RBC concentrations in the infection of *An. aquasalis* with *P. viva*x. To our knowledge, there is no such information about similar studies developed with *P. vivax* in a natural mosquito vector. We sought to characterize the infection of the vector *via* infective blood meals containing chitinase, trypsin inhibitor and hematocrits at different concentrations. Detailed study interrupting the development of the PM associated to the inhibition of the proteolytic activity and the addition of distinct RBC concentrations during the infection of a natural vector by *P. vivax* was expected to clarify if these factors affect its vector competence or not. This study established that there is a balance between the PM role, RBC concentration and digestive enzyme activity influencing the establishment and development of *P. vivax* modulating the infection inside the New World malaria vector *An. aquasalis*.

## Methods

### *Anopheles aquasalis* rearing and maintenance

Mosquitoes were reared at the insectaries of the Laboratory of Medical Entomology at the Fundação de Medicina Tropical Dr Heitor Vieira Dourado (FMT-HVD), Manaus, and at the Instituto de Pesquisas René Rachou (Fiocruz-MG), Belo Horizonte, Brazil. Colonies were kept between 24–26 °C and 70–80% of relative humidity on a 12:12 h light-dark cycle. Larvae were hatched in room temperature water and ground TetraMin® fish food (Madison, Wisconsin, USA) was provided daily. Larvae were allowed to pupate and emerge into adults in an enclosed mesh-covered cage with water and fed *ad libitum* in 10% sucrose solution until two days before the infective blood meals [11].

### Blood collection and ethics statement

Adult volunteers (aged > 18 years) residing in the region of Manaus, presenting at the FMT-HVD with microscopically confirmed *P. vivax* malaria, were invited to participate in the study. About 3 ml of blood were collected by venipuncture and placed into a sterile heparinized vacutainer tube.

### Midgut dissection, histology and scanning electron microscopy (SEM)

In order to observe the structure and the chitinase effect on the PM inside the midgut of *An. aquasalis*, two blood-feeding experiments were performed: (i) feeding with normal blood meal consisting of a blood sample of non-infected health donors (control group) and (ii) feeding with normal blood meal mixed with 1 U/ml *Streptomyces* chitinase (chitinase-treated group) as described elsewhere [23, 25, 29, 30]. The blood meals were offered to groups of *An. aquasalis* for 30 min through membrane feeders, as described previously [11, 12]. Control and chitinase-treated mosquito groups were dissected and processed 24 h after the blood meal for histology or for SEM. Mosquitoes were immobilized at 4 °C and transferred to a Petri dish placed on ice. In order to conduct morphological observation, midguts were dissected in phosphate-buffered saline (PBS), pH 7.4 under a stereomicroscope. These midguts were fixed at room temperature with 2.5% glutaraldehyde (GA) solution in 0.1 M caccodylate buffer and after 24 h were routinely processed for histology or SEM. For histological observation, midguts were dehydrated and embedded in Historesin (Leica®, Wetzlar, Germany). 2-μm histological sections were obtained, stained with 1% toluidine blue solution for 3 min and mounted on glass slides to be analyzed and photographed under optical microscope [23, 30]. For SEM observation, the GA-fixed midguts were post-fixed with 1% osmium tetroxide solution containing 0.8% potassium ferricyanide for 2 h. Next, they were dehydrated in serial crescent acetone solution and processed in a CO_2_ critical point device and assembled on stubs. The dried midguts were fractured with entomological stilettos and metallized with 20 nm gold particles to be observed and analyzed in a scanning electron microscope [31]. Histology and SEM were used to observe morphological aspects of the mosquito’s PM.

### Oral *P. vivax* infection of *An. aquasalis*

Adult mosquitoes were sugar starved overnight prior to infection *via* membrane-feeding assays. Briefly, *P. vivax* infected blood samples in diverse conditions, as described below, were offered to groups of 50 to 200 *An. aquasalis* for 30 min through membrane feeders at 37 °C, as described previously [11, 12]. Two sets of experiments were performed to study the PM role and the effect of RBC concentration in the mosquito infection by *P. vivax*.

To evaluate the PM role, blood meals of *P. vivax*-infected blood added with distinct substances were offered to the four different groups of *An. aquasalis* mosquitoes according to the addition of chitinase or soybean trypsin inhibitor: (i) Control (infected blood meal); (ii) Chitinase (infected blood samples plus 1 U/ml *Streptomyces griseus* chitinase); (iii) Trypsin inhibitor (infected blood samples plus 1 U/ml soybean trypsin inhibitor); and (iv) Chitinase + Trypsin inhibitor (infected blood samples plus chitinase and soybean trypsin inhibitor). All the infected blood samples that compose each experimental group were collected from the same patient.

Chitinase and trypsin inhibitor concentrations used were as previously described [23, 25, 31–33]. All reagents were from Sigma-Aldrich® (Saint Louis, USA). Biological replicates were performed for each experimental condition.

To assess the effect of different RBC concentrations (distinct hematocrits) on *An. aquasalis* infection, patient blood samples were centrifuged (2000× *g* for 15 min) and after removal of plasma, *P. vivax*-infected erythrocytes were enriched by Percoll gradient or magnetic column as previously described [34]. The enriched infected cells were divided equally in three parts and used to prepare 1 ml of blood meal offered to three different groups of mosquitoes according to RBC concentrations: (i) 40% hematocrit: 400 μl of non-infected RBC and 600 μl of non-immune AB serum (control group); (ii) 30% hematocrit: 300 μl of non-infected RBC and 700 μl of non-immune AB serum; and (iii) 15% hematocrit: 150 μl of non-infected RBC and 850 μl of non-immune AB serum. Therefore, the final gametocyte counts were the same for the 3 experimental groups. After the blood-feeding experiments, only fully engorged mosquitoes were transferred to rearing containers. They were maintained in the insectary to be dissected at appropriated times after the blood meal for processing as described below. Biological replicates were performed for each experimental condition.

### Analyses of *P. vivax* infection of *An. aquasalis*

Nine days after infective blood meals, midguts from all experimentally infected mosquito groups were dissected in PBS as described above, stained with 0.1% commercial Mercurochrome (Merbromin®, Saint Louis, USA), placed under a cover glass and examined for the presence of oocysts in an optical microscope. The infection rate (percentage of infected mosquito midguts) and the infection intensity (mean number of oocysts per midgut) on each mosquito were recorded and compared among the experimental groups.

### Statistical analysis

Shapiro-Wilk test was used to verify the normality of the distribution. Multiple-sample comparisons were analyzed using one-way ANOVA. A nonparametric Kruskal-Wallis test with Dunn’s multiple comparisons *post-hoc* test was used for intensity analysis, and for the infection rate with parametric distribution the ordinary one-way ANOVA with Turkey’s multiple comparisons *post-hoc* test. Significant correlations with *P* values ≤ 0.05 were considered significant. All statistical analyses were performed using GraphPad Prism® software (Prism 5.01; GraphPad Software Inc.).

## Results

### Structure of *An. aquasalis* PM and the effect of the exogenous chitinase

**Fig. 1:**
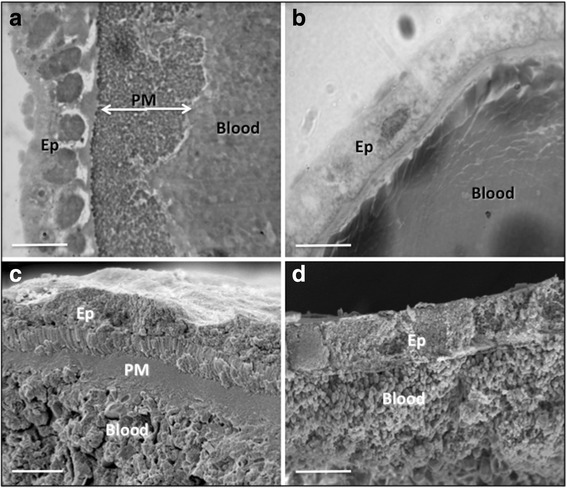
Morphology of *An. aquasalis* midguts after normal and chitinase-containing blood meals. Histology (**a**) and SEM (**c**) of *An. aquasalis* midguts 24 h after a normal blood meal. The thick PM is visible isolating the midgut epithelium from the partially digested blood meals. Similar histology (**b**) and SEM (**d**) of *An. aquasalis* midguts 24 h after a chitinase-containing blood meal. The PM is absent inside the midgut, and the blood meal is in direct contact with the epithelium. *Abbreviations*: PM, peritrophic matrix; Ep, epithelium; Blood, blood meal. *Scale-bars*: a-d, 50 μm

The *An. aquasalis* mosquito midgut 24 h after the normal blood meal (control group) revealed a well-developed PM as a coarse-thick and dense structure underneath the single midgut epithelium (Fig. 1a, c). At this time point, the PM is enclosed just above the midgut epithelium, segregating the entire blood meal. In contrast, *An. aquasalis* midguts 24 h after the chitinase-containing blood meal showed a total absence of the PM, with the partially digested blood meal in direct contact with the midgut epithelium (Fig. 1b, d).

### PM role and digestive enzymes in *P. vivax* infection of *An. aquasalis*

**Fig. 2:**
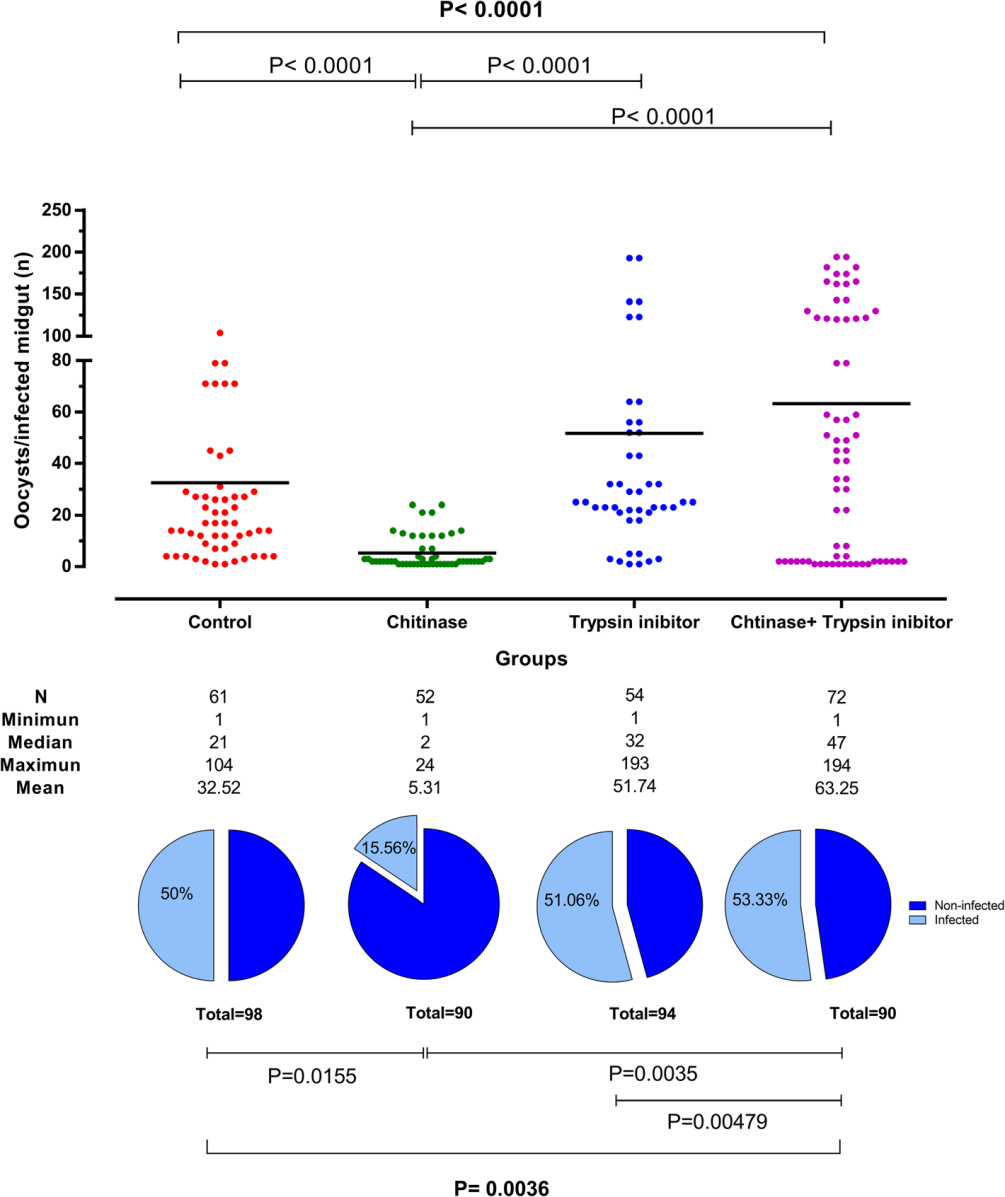
Effect of exogenous chitinase and trypsin enzyme inhibitor on the infection of *Anopheles aquasalis* with *Plasmodium vivax.* The intensity of infection of each experimental group is presented in the top graph as the oocyst number per midgut (dots), the black lines represent the mean (Kruskal-Wallis H-test: *χ*^2^ = 41.333, *df* = 3, *P* < 0.0001). The infection rate is represented in the bottom graph as the percentage of *P. vivax* infected mosquitoes (light blue pie section) (ANOVA: *F*_(3, 8)_ = 10.63, *P* = 0.0036)

The roles of chitinase and trypsin in *P. vivax* infection of *An. aquasalis* were analyzed by calculating the infection rate and intensity in different groups of treated/untreated mosquitoes. All experimental groups showed infected *P. vivax* mosquitoes but with distinct infection rates (ANOVA: *F*_(3, 8)_ = 10.63, *P* = 0.0036) and infection intensities (Kruskal-Wallis H-test: *χ*^2^ = 41.333, *df* = 3, *P* < 0.0001) (Fig. 2). Trypsin inhibitor group presented similar infection rate (*P* = 0.1672) and infection intensities (*P* = 0.2059). The Chitinase group showed a significant reduction in infection rate (*P* = 0.0155) and infection intensity (*P* < 0.001) compared to Control group. On the other hand, the Chitinase + Trypsin group presented a similar infection rate (*P* = 0.660) and infection intensity (*P* > 0.999) to those of Control group, and higher infection rate (*P* = 0. 0035) and infection intensity (*P* < 0.001) when compared with the Chitinase group.

### Effect of RBC concentration in the *P. vivax* infection of *An. aquasalis*

**Fig. 3:**
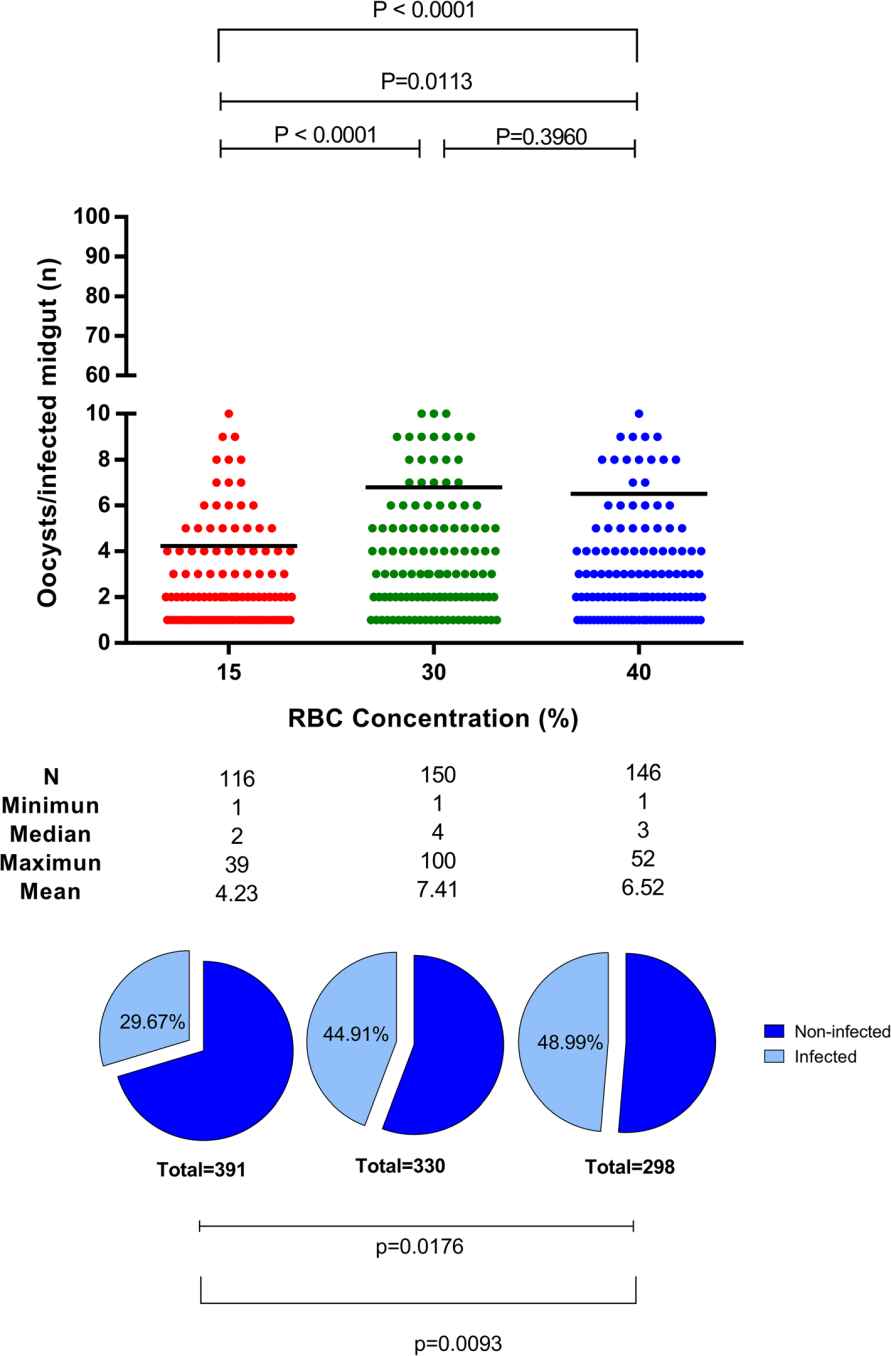
Effect of blood meal hematocrit (RBC concentration) on the infection of *An. aquasalis* with *P. vivax.* The intensity of infection of each experimental group (red 15% hematocrit, green 30% hematocrit, blue 40% hematocrit) is presented in the top graph as the oocyst number per midgut (dots), the black lines represent the mean (Kruskal-Wallis H-test: *χ*^2^ = 19.090, *df* = 2, *P* < 0.0001). The infection rate is represented in the bottom graph as the percentage of *P. vivax* infected mosquitos (light blue pie section) (Kruskal-Wallis H-test: *χ*^2^ = 8.578, *df* = 2, *P* = 0.0093)

The role of RBC in *P. vivax* infection of *An. aquasalis* was analyzed by determination of the infection rate and intensity in groups of mosquitoes fed with the same amount of *P. vivax* gametocytes diluted in different concentration hematocrits (15%, 30% and 40%). All experimental groups showed infected *P. vivax* mosquitoes but with distinct infection rates (Kruskal-Wallis H-test: *χ*^2^ = 8.578, *df* = 2, *P* = 0.0093) and/or infection intensities (Kruskal-Wallis H-test: *χ*^2^ = 19.090, *df* = 2, *P* < 0.0001) (Fig. 3). The 40% hematocrit (normal RBC) group of mosquitoes presented an infection rate of 48.9% and an infection intensity of 6.5 oocysts/infected midgut; whereas the 15% hematocrit (lowest RBC) group of mosquitoes showed a reduction in infection rate (29.7%) and intensity of 4.2 oocysts/infected midgut (*P* = 0.0174 and *P* = 0.011, respectively). Moreover, the 30% hematocrit (intermediate RBC concentration) group presented a similar infection rate (44.9%) and infection intensity of 7.4 oocysts/infected midgut to 40% hematocrit group (*P* > 0.999 and *P* = 0.396) and higher infection intensity than the 15% hematocrit group (*P* < 0.001).

## Discussion

There is a significant knowledge gap about malarial infections by mosquito vectors from the American continent compared to vector-parasite pairs of African and Asian countries*.* Furthermore, most interaction studies were developed with the human malarial species *P. falciparum* or with avian and murine *Plasmodium* species. Only recently have interaction studies addressed interaction between Amazonian vectors and *P. vivax*, the most prevalent human parasite causing malaria in the New World. These studies have revealed important aspects of infection by Old World vectors [12].

The PM of mosquitoes acts as a physiological and physical barrier with several functions inside the midgut, including preventing tissue damage, controlling the digestive enzyme secretion and regulating pathogen infection as a vector response to either establishing or not a persistent infection. Some substances added to the ingested blood meal may disturb or even prevent PM formation. Since chitin is a main component of the PM structure, addition of exogenous chitinase has been demonstrated to prevent PM formation in blood-fed mosquitoes and other Diptera [25, 30–33].

Chitinase is a natural product synthesized by many organisms, especially fungi, which plays a key role in destabilization of chitinous structures, such as PM, or degradation of chitinous structures produced by the pathogen itself or its host [35]. For example, addition of chitinase from *S. griseus* to the blood meal offered to the sand fly *Lutzomyia longipalpis* [31] and to the mosquito *Aedes aegypti* [32] (vectors of *Leishmania* and important arboviruses, respectively) resulted in the absence of PM. Similarly, the histological and SEM observations presented here demonstrated that *An. aquasalis* mosquitoes that ingested *S. griseus* chitinase did not synthetize PM and, consequently, the mosquito midgut epithelium was in direct contact with the blood meal. Based on this initial knowledge, a series of experiments were developed in order to understand the role of the PM in *P. vivax* infection and the influence of the trypsin inhibitor on the American vector *An. aquasalis*, including comparing its role in other malarial vector-parasite pairs.

*Plasmodium* species causative agents of murine and avian malaria have been used as models to understand the PM role in mosquito vectors. Development of *P. berghei* infection, a murine malarial parasite, in *An. stephensi* was unaffected by the absence of PM induced by chitinase ingestion [28]. However, preventing PM formation in *Ae*. *aegypti* by knocking down chitin synthase caused a lower infection rate by *P. gallinaceum*, an avian malarial parasite [29]. Moreover, increasing the thickness of the *Ae. aegypti* PM due to double blood-feedings only reduced the infection rate of *P. gallinaceum* [28]. This infection was completely blocked when ingestion of the chitinase inhibitor allosamidin resulted in a thicker PM [25]. Curiously, the same authors observed that the absence of PM resulting from ingestion of chitinase or polyoxin D did not affect *P. gallinaceum* infection [36]. These results suggest that in murine and avian malarial parasites the PM may act as a partial, but not absolute, barrier to parasite invasion of the mosquito midgut in distinct vector-*Plasmodium* pairs. Nevertheless, the present study demonstrated that the absence of the PM in the vector *An. aquasalis* caused significant reduction in *P. vivax* infection. This fact emphasizes the importance of the PM role in the establishment of *P. vivax* infection in a relevant American vector.

One important PM role is controlling the flux of digestive enzymes produced by the mosquito midgut epithelium; it appears that PM limits the rate of digestion by reducing the diffusion of hydrolytic enzymes [37]. In Diptera, the porous PM induces a gradient of digestive enzyme activity in the blood meal inside the midgut, causing stronger digestive actions in the periphery than in the center of the digestive bolus [29, 31, 38–40]. The mosquito digestive tract contains a complex set of endoproteases and exopeptidases [41]; the major endoprotease acting in the blood meal digestion of mosquitoes is trypsin [42]. Proteolytic enzymes in mosquito midguts are located along the periphery of the blood bolus during the first 24 h after blood feeding [29]. Abraham & Jacobs-Lorena [43] suggested that *Plasmodium* ookinetes in the outer parts of the blood meal, close to PM, die first due to the action of digestive enzymes, whereas ookinetes located closer to the interior of the blood meal, and consequently farther away from the effects of these enzymes, have a longer time to differentiate and survive enzymatic action. Interestingly, one of the first conclusive PM roles in protecting parasites has been reported in the sand fly vector *Phlebotomus papatasi*, infected with *Leishmania major* [31]. Although PM plays an important role in protecting the sand fly, it also creates a barrier that protects *L. major* from the action of digestive enzymes by limiting exposure of the parasite during first 24 h when it is deeply located in the center of the blood meal and vulnerable to proteolytic damage. In *An. aquasalis*, trypsin activity peaked between 12–24 h after blood-feeding, similar to other anophelines [43, 44], and in the same period when ingested *P. vivax* are transformed into motile forms, the ookinetes, in order to cross the PM and invade the midgut epithelium. Actually, gametes, zygotes and early non-differentiated ookinetes, the first *Plasmodium* forms inside the mosquito vectors in the first 24 h of infection, are very sensitive to digestive enzymes [22, 24]. The PM role is to provide a favorable midgut environment for *Plasmodium* survival and infection development. This study showed that the association of chitinase with trypsin inhibitor restored *P. vivax* infection rates to levels similar to that of mosquitoes that have a structured PM but with higher infection intensity, showing numerous oocysts in the mosquito midgut. Only the ingestion of trypsin inhibitor by non-chitinase treated mosquitoes resulted in a higher infection intensity. Indeed, these results suggest that the PM acts with enzyme digestive activities to regulate infection of *P. vivax* in *An. aquasalis*.

The RBC concentration in the blood meal ingested by hematophagous insects, including mosquitoes, affects the formation dynamics of the PM and, consequently, pathogen development in the vector [23, 45]. *Anopheles stephensi* and *Ae. aegypti* that are fully engorged with large blood meals in the midgut have a thicker, more well-developed PM than mosquitoes fed partially [46]. Also, there is a linear correlation between RBC concentration of the ingested infected blood meal and infectivity of *Aedes aegypti* by *P. gallinaceum* [45].

Anemia is a common feature in malaria infections [24]; moreover, a recent study revealed that moderate anemia increases the probability of vivax malaria patients to carry gametocytes [46]. In anophelines, the RBC component of the meal is concentrated by expelling plasma whilst feeding, a process termed prediuresis [47] that increases the number of infected cells ingested [48], thereby enhancing parasite transmission. Taylor & Hurd [49] demonstrated using *P. yoelii* and *An. stephensi* that mosquitoes are unable to compensate for large decreases in RBC concentration by prediuresis, but a slight decrease in host hematocrit promoted a higher hemoglobin content in the vector, therefore a higher amount of erythrocytes uptake.

Our study demonstrated that very low RBC concentration diminishes *P. vivax* ability to establish infection in *An. aquasalis*. Additionally, we showed that a moderate decrease in hematocrit (30%) did not alter *P. vivax* infectivity to the vector. It is important to highlight that the three mosquito groups fed with different RBC concentrations ingested the same amount of gametocytes per ml of blood meal. Nevertheless, the lower hematocrit (15%) had a lower infection and intensity rates. Thus, RBC concentration modulates *P. vivax* infection. One possible explanation is the thicker, well-developed PM in *An. aquasalis* fed with high hematocrits, as described for *Aedes aegypti* infected with *P. gallinaceum* by [45]. Also, the dispersion and proximity of digestive enzymes have deleterious effects on the first *Plasmodium* forms, since the food bolus is small in the mosquitoes fed with low hematocrit.

## Conclusions

An enhanced knowledge of *P. vivax* development inside the New World malaria vector *An. aquasalis* is critical for future strategies aimed at blocking parasite development. This study established that there is a balance between the PM role, RBC concentration and digestive enzyme activity, influencing the establishment and development of *P. vivax* inside *An. aquasalis*. One hypothesis is that the absence of PM, as well as a low RBC concentration, probably facilitate digestive enzyme dispersion throughout the blood meal inside the mosquito midgut causing direct damage to *P. vivax*. On the other hand, a high RBC concentration supports a thick well-developed PM and protects *P. vivax* from being killed by digestive enzymes inside the *An. aquasalis* midgut. Thus, here we provide new understanding of the mechanisms by which New World mosquito vector limit the development of *P. vivax*, which may lead to new methods for controlling malaria. Further studies of this complex system may provide insights into other details of the malaria vector response to infection.

## Abbreviations

FMT-HVD: Fundação de Medicina Tropical Dr Heitor Vieira Dourado
GA: glutaraldehyde
PBS: phosphate-buffered saline
PM: peritrophic matrix
RBC: red blood cells
SEM: scanning electron microscopy
